# Editorial: Recent advances in mitochondria-associated endoplasmic reticulum membranes (MAMs) in heart-related diseases

**DOI:** 10.3389/fcvm.2023.1168152

**Published:** 2023-03-16

**Authors:** Yang Yang, Yi Luan

**Affiliations:** Clinical Systems Biology Research Laboratories, Translational Medicine Center, The First Affiliated Hospital of Zhengzhou University, Zhengzhou, China

**Keywords:** CVDs (Cardio vascular diseases), mitochondria, endoplasmic recticulum, ROS, mitochondria-associated endoplasmic reticulum membranes

**Editorial on the Research Topic** Recent advances in mitochondria-associated endoplasmic reticulum membranes (MAMs) in heart-related diseases

Mitochondria-associated endoplasmic reticulum membranes (MAMs) are the physical connections between the two important organelles of the endoplasmic reticulum and mitochondria, and also the key to the normal functioning of the endoplasmic reticulum and mitochondria (1). It enables Ca^2+^ to be efficiently transported from the endoplasmic reticulum to the mitochondria, and is involved in the regulation of ROS, lipid metabolism, cell apoptosis, etc., so as to maintain cell metabolism and normal function (2). The concept of MAMs was proposed by John Ruby et al. as early as 1969 and was not isolated from rat livers until 1990 by Jean Vance (3). In the following 30 years, with the development of electron microscopy, high resolution imaging and other technologies, people have a clear and multi-level understanding of the important functions of MAMs.

By mass spectrometry, more than 1,000 proteins have been found to be concentrated in MAMs structures, and many of these proteins have been found to affect the progression of cardiovascular diseases. Studies have shown that MAMs play an important role in the development of cardiovascular diseases such as heart failure, myocardial ischemia-reperfusion injury, and atherosclerosis. In this research topic, Yi Luan and Yu Zhang et al. discussed key MAMs-associated proteins and their functions in cardiovascular system and defined their roles in the progression of cardiac hypertrophy and heart failure (Luan et al.; Zhang et al.). Jiahao Zhao et al. showed *the role of mitochondria-associated membranes mediated ROS on NLRP3 inflammasome in CVDs* (Zhao et al.). Importantly, the NLRP3 inflammasome has been shown to be influenced by MAMs to affect various cellular functions and, with it, CVDs (1).

Interactions between organelles can be involved in the progression of various cardiovascular diseases by influencing cell function (4). For example, MAMs mediated Ca^2+^ transport to mitochondria affects energy metabolism of cardiovascular cells, and is associated with myocardial hypertrophy, phenotypic changes and proliferation of VSMCs (5). MAMs mediated calcium flow also induces hypertrophic signal transduction and contraction responses; MAMs also induce endoplasmic reticulum and mitochondria-related cellular processes, such as ER stress and apoptosis, by regulating lipid and calcium ion transfer (6). MAMs recruit key signaling molecules and effector proteins as platforms for dynamic changes in inflammasome and mitochondria (1). Multiple studies have demonstrated that MAMs mediated mitochondrial function changes have occurred in the early stages of cardiovascular disease. Therefore, intervention for MAMs at the early stage of CVDs is also a new idea to treat CVDs.

Understanding the regulatory mechanisms and targeting proteins of MAMs integrity maintenance is important to explore new therapeutic targets for the prevention or treatment of heart-related diseases. For example, fluvoxamine has been used to treat heart failure and cardiac dysfunction in mouse and rat models of transverse aortic contraction due to its high affinity for Sig1R (7). Acetylcysteine, moreover, as a kind of antioxidant, is used in the treatment of mitochondrial damage and muscle dysfunction, this suggests that ROS are involved (8).

This research topic describes in detail the function of MAMs structure and its important role in cardiovascular diseases. On the one hand, MAMs maintain cell homeostasis and play a protective role by promoting mitochondrial division and mitochondrial autophagy; on the other hand, the increase of MAMs structure causes excessive mitochondrial division, mitochondrial calcium overload or oxidative stress. It causes pathological damage to cells. However, there are still many questions worth studying, such as whether the regulation effect of MAMs on cell function is similar in different tissues and conditions. What are the key molecules that maintain the structure and function of MAMs and how? Do mitochondria also influence endoplasmic reticulum function through MAMs? In conclusion, understanding the mechanisms that regulate the formation of MAMs structures and identifying the functions of the proteins involved in MAMs structures will help to suggest new strategies for the prevention and treatment of cardiovascular diseases in the future.
